# Prospective surveillance of healthcare associated infections in a Cambodian pediatric hospital

**DOI:** 10.1186/s13756-017-0172-5

**Published:** 2017-01-23

**Authors:** Pasco Hearn, Thyl Miliya, Soklin Seng, Chanpheaktra Ngoun, Nicholas P. J. Day, Yoel Lubell, Claudia Turner, Paul Turner

**Affiliations:** 1 Cambodia-Oxford Medical Research Unit, Microbiology Department, Angkor Hospital for Children, PO Box 50, Siem Reap, Cambodia; 2 Centre for Tropical Medicine and Global Health, Nuffield Department of Medicine, University of Oxford, Oxford, UK; 3 Angkor Hospital for Children, Siem Reap, Cambodia; 4 Mahidol-Oxford Tropical Medicine Research Unit, Faculty of Tropical Medicine, Mahidol University, Bangkok, Thailand

**Keywords:** Healthcare associated infection, Prospective HAI surveillance, Pediatric HAI, Cambodia

## Abstract

**Background:**

Healthcare associated infections (HAI) are the most common preventable adverse events following admission to healthcare facilities. Data from low-income countries are scarce. We sought to prospectively define HAI incidence at Angkor Hospital for Children (AHC), a Cambodian pediatric referral hospital.

**Methods:**

Prospective HAI surveillance was introduced for medical admissions to AHC. Cases were identified on daily ward rounds and confirmed using locally adapted Centers for Disease Control and Prevention (CDC) definitions. During the surveillance period, established infection prevention and control (IPC) activities continued, including hand hygiene surveillance. In addition, antimicrobial stewardship practices such as the creation of an antimicrobial guideline smartphone app were introduced.

**Results:**

Between 1st January and 31st December 2015 there were 3,263 medical admissions and 102 HAI cases. The incidence of HAI was 4.6/1,000 patient-days (95% confidence interval 3.8–5.6) and rates were highest amongst neonates. Median length of stay was significantly longer in HAI cases: 25 days versus 5 days for non-HAI cases (*p* < 0.0001). All-cause in-hospital mortality increased from 2.0 to 16.1% with HAI (*p* < 0.0001). Respiratory infections were the most common HAI (54/102; 52.9%). Amongst culture positive infections, Gram-negative organisms predominated (13/16; 81.3%). Resistance to third generation cephalosporins was common, supporting the use of more expensive carbapenem drugs empirically in HAI cases. The total cost of treatment for all 102 HCAI cases combined, based on additional inpatient days, was estimated to be $299,608.

**Conclusions:**

Prospective HAI surveillance can form part of routine practice in low-income healthcare settings. HAI incidence at AHC was relatively low, but human and financial costs remained high due to increased carbapenem use, prolonged admissions and higher mortality rates.

**Electronic supplementary material:**

The online version of this article (doi:10.1186/s13756-017-0172-5) contains supplementary material, which is available to authorized users.

## Background

The most common, preventable adverse event following admission to a healthcare facility is a healthcare associated infection (HAI). Such infections are now a major cause of morbidity and mortality worldwide [1, 2]. Admissions are often prolonged by HAI, whilst their association with increasingly resistant bacteria requires that more expensive antimicrobials are often employed. As such, HAI represent an ever growing human and financial cost in both high and low-income settings, but estimating the scale of the issue is problematic.

The World Health Organization (WHO) report on the global burden of HAI in 2011 stated that “there is an urgent need to establish reliable systems for HAI surveillance and to gather data on the actual burden on a regular basis” [3]. Pooled information from high-income countries suggested an overall HAI prevalence of 7.6 per 100 admissions and an incidence of 17.0 per 1,000 patient-days [3]. Few middle and low-income countries carry out HAI surveillance, but pooled data from such studies show a significantly higher prevalence of 15.5 per 100 admissions and incidence of 47 · 9 per 1,000 intensive care unit (ICU)-days [4]. It is estimated that 4.5 million HAI episodes occur each year in Europe, costing around €7 billion [5]. Estimating costs in low-income settings is very challenging due to the variety of settings, standards of medical care and lack of data. Nevertheless, an extended length of stay (LOS) of 5–29.5 days per HAI and an excess mortality of 18–29.5% [3] are likely to represent significant burdens for these countries.

Limited resources are available to carry out prospective studies on HAI [6]. Much of the available data come from adult populations within high-income countries. Accurate comparisons between healthcare systems have been facilitated by the introduction of clear case definitions [7–9], but these are centered on adult infections. The risks of HAI are different for children, as are their clinical presentations [10]. Children have immature immune systems, increased susceptibility to viral infections and frequently present without fever, making the diagnosis of HAI more challenging [11]. Such factors highlight the need for more consistent approaches to HAI surveillance, particularly in pediatric populations.

Reductions in HAI rates of 20 to 30% have been seen with the introduction of effective infection prevention and control (IPC) programs, alongside HAI surveillance [12–14]. Other interventions known to reduce HAI rates include written guidelines; organization of IPC at a hospital level; education and training, especially when accompanied by audit and feedback; and surgical instrument sterilization procedures [15–17]. Lack of resources, however, requires prioritization of the most cost-effective methods. Poor hand hygiene is widely accepted as the most important risk factor for HAI [2, 18, 19]. The International Nosocomial Infection Control Consortium (INICC) has repeatedly shown, in resource-poor settings, a comprehensive and multifaceted approach can improve hand hygiene compliance by up to 48% [20–23]. Focusing on antimicrobial stewardship in addition to hand hygiene has also been shown to improve HAI rates in low-income settings [24].

In Cambodia, a low-income Southeast Asian country, there are very limited data on the burden of HAI. Stoesser et al. reported previously the effects of introducing an IPC program to Angkor Hospital for Children (AHC). Hand hygiene surveillance and a ventilator-associated pneumonia (VAP) care bundle were introduced in 2010 and the impact measured by regular HAI point prevalence survey (PPS). During the study period, hand hygiene compliance doubled to 51.6%. A significant reduction in HAI prevalence was also noted, from a median of 15.8% in the first half of the year to 11.1% in the second [25].

However, the lack of prospective, incidence-based surveillance means that the true burden of pediatric HAI in low-income countries is not well understood. Recognized approaches to HAI reduction are also more challenging where resources are limited. The aim of this surveillance program was to prospectively define the incidence of HAI in medical admissions to AHC during a period of ongoing IPC activity and increased antimicrobial stewardship. Once established into routine care, ongoing prospective surveillance will allow the assessment of future IPC interventions to occur in real time.

## Methods

### Surveillance site

AHC is located in Siem Reap, the capital town of Siem Reap province, northwest Cambodia. According to the 2008 national census, the province had a total population of 896,443, including 322,857 children aged <15 years [26]. AHC is a non-governmental organization and one of two pediatric hospitals located in the province. This 89 bedded hospital provides secondary and tertiary level care to children aged <16 years with no geographic restriction. There are approximately 160,000 patient presentations and over 4,000 admissions per year. In addition to medical and surgical wards, there is an ICU, neonatal intensive care unit (NICU) and special care baby unit (SCBU).

The microbiology department at AHC provides microscopy, culture and antimicrobial sensitivity results to clinicians on a range of samples, along with treatment advice where required. The laboratory follows guidance from the Clinical & Laboratory Standards Institute (CLSI) for antimicrobial susceptibility testing and participates in internal and external quality assurance programs (Pacific Paramedical Training Centre). The clinical microbiologists, having trained as physicians prior to specializing in infection, regularly discuss and review patients and their treatment on the wards.

### IPC and antimicrobial stewardship activities

IPC activities started at AHC in 2010 and include regular hand hygiene surveillance, monthly multidisciplinary IPC meetings, quarterly PPS and an annual staff training course. Compliance with the first moment of the WHO guidelines on hand hygiene is monitored and rates fed back to ward representatives at monthly meetings. Education focusses on improving hand hygiene, maintaining a clean clinical environment and minimizing the duration of exposure to major HAI risk factors, such as urinary catheters, central venous catheters and ventilation. The IPC program uses the WHO-supported Cambodian national IPC guideline as a reference point [27].

Infection and antimicrobial stewardship ward rounds started on a weekly basis on medical wards and twice weekly on ICU. These were attended by medical students, junior and senior doctors and focused on infection cases within each ward.

Pre-existing hospital antimicrobial guidelines were updated and converted into a free smartphone app (“MicroGuide” [28]), also available on ward computers. The app included local and national guidelines where available and appropriate international guidelines where they were not. In addition to diagnostic and treatment algorithms, the app contained HAI definitions and details about our surveillance. A survey of doctors at AHC was conducted one month after release of the app to assess its ease of use and utility in daily practice. Use of the app was also monitored throughout by its producers. Four continued medical education (CME) sessions for all clinical staff were used to introduce the “MicroGuide” app, to discuss increasing antimicrobial resistance, the benefits of antimicrobial stewardship and the purpose of monitoring HAI rates.

Quarterly PPS continued, with an additional appropriateness measure, based on the assessment of a clinical microbiologist. A prescription was deemed inappropriate if the choice of antimicrobial deviated from the guidelines for the documented diagnosis; if the dose was not as per guidelines and according to the patient’s weight; if the duration was longer than the guidelines suggested, without clearly documented reason.

### Prospective HAI surveillance

Daily ward rounds started on medical, ICU, NICU and SCBU. Discussion with the attending team enabled HAI case-finding five days per week, with weekend cases identified each Monday.

HAI were considered possible in patients admitted for at least 48 h, or if presenting with likely infection following discharge from AHC within the preceding 48 h. Cases were confirmed and categorized by syndrome using locally adapted CDC definitions (see Additional file 1). “Possible HAI” cases were those given a clinical diagnosis of HAI and treatment to reflect this, but who did not satisfy the criteria for any HAI syndromes.

Case report forms recorded patient demographics, reasons for admission, risk factors for HAI, symptoms, signs and key investigation results. The medical team was offered management advice, but investigations and final decisions were made at the discretion of the clinicians. Treatment, admission duration and outcome data were recorded and reported at the end of each calendar month to heads of department within the hospital.

### Data management and analysis

Following case identification, patient data were anonymized and entered into a password protected database (Access 2013, Microsoft, Redmond, WA). Data were analyzed using the R statistical package, version 3.2.0 [29]. HAI incidence was determined as the number of cases per 1,000 patient-days. For comparison within the literature the ‘attack rate’ was also calculated as the number of HAI cases per 100 admissions. Poisson confidence intervals were calculated for incidence rates. Denominator information was extracted from the hospital electronic database which documents all admission and discharge dates for all in-patients. Each case was matched by age and admission ward with two controls and comparisons between groups were made using the Wilcoxon Rank Sum test for continuous variables and the Chi-squared test for proportions. Two-sided *p*-values of < 0.05 were considered an indication of statistical significance.

### HAI costings

The impact of HAI on LOS was estimated by comparing each HAI case with two non-HAI controls, matched by both age-group and admission-ward, as a correlate for severity at presentation. Costings from a complete top-down economic analysis of AHC in 2011 were inflation-adjusted to reflect current prices [30]. This produced a cost per patient-day of $130 on the medical ward/SCBU and $184.50 on ICU/NICU. These figures were then combined with the additional LOS data to estimate overall HAI costs. As a separate calculation, the cost of antimicrobial usage in HAI cases was calculated using pharmacy pricing records for each drug prescribed, assuming single drug vials were used per dose.

## Results

There were 4,300 admissions to AHC during 2015. Surgical admissions, where surveillance was not active, were removed from analysis, along with those admitted for <48 h. A total of 3,263 (76%) medical admissions were followed until discharge, representing 21,995 patient-days. The most frequent reasons for admission were pneumonia, gastroenteritis and dengue, followed by acute bronchiolitis and asthma.

**Fig. 1:**
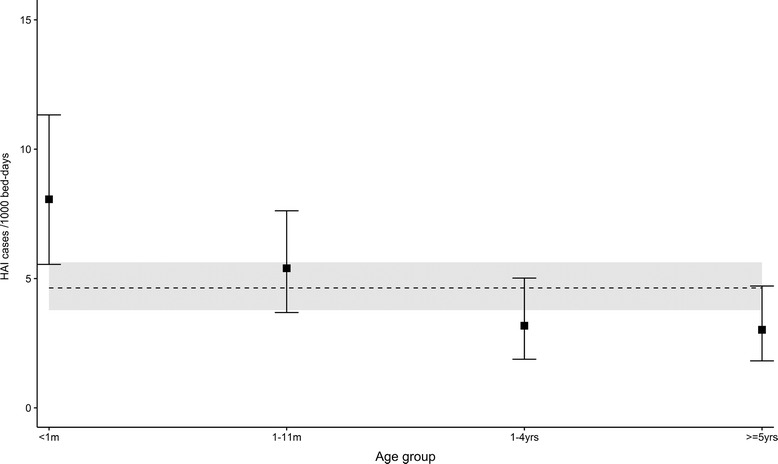
HAI incidence and 95% CI by age group (dashed line showing overall annual incidence with 95% CI) *m* months of age, *yr* years of age

**Fig. 2:**
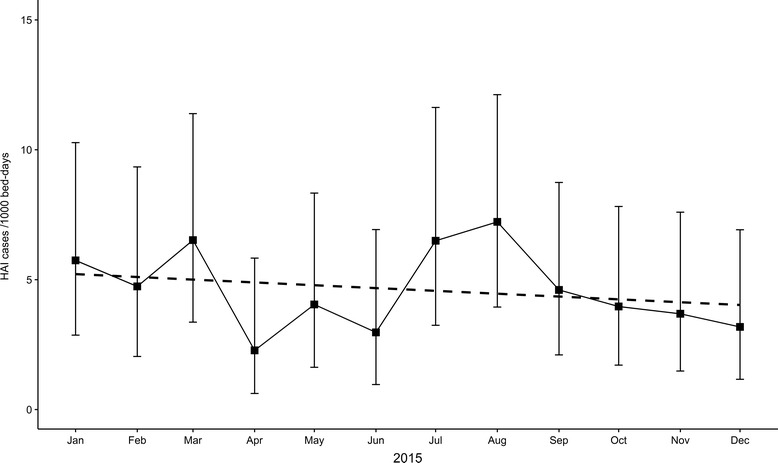
HAI incidence by month during 2015 (dashed line showing linear trend)

One hundred two HAI episodes were identified from 93 separate admissions, resulting in an annual HAI incidence of 4.6 per 1,000 patient-days (95% confidence interval (CI) 3.8–5.6) and an attack rate of 3.1 per 100 admissions (95% CI 2.5–3.8). Incidence varied by age group, with the majority of infections affecting those under one year of age (Fig. 1). Over the course of the surveillance period there was a downward trend in HAI incidence. This was not, however, found to be statistically significant (Fig. 2).

**Fig. 3:**
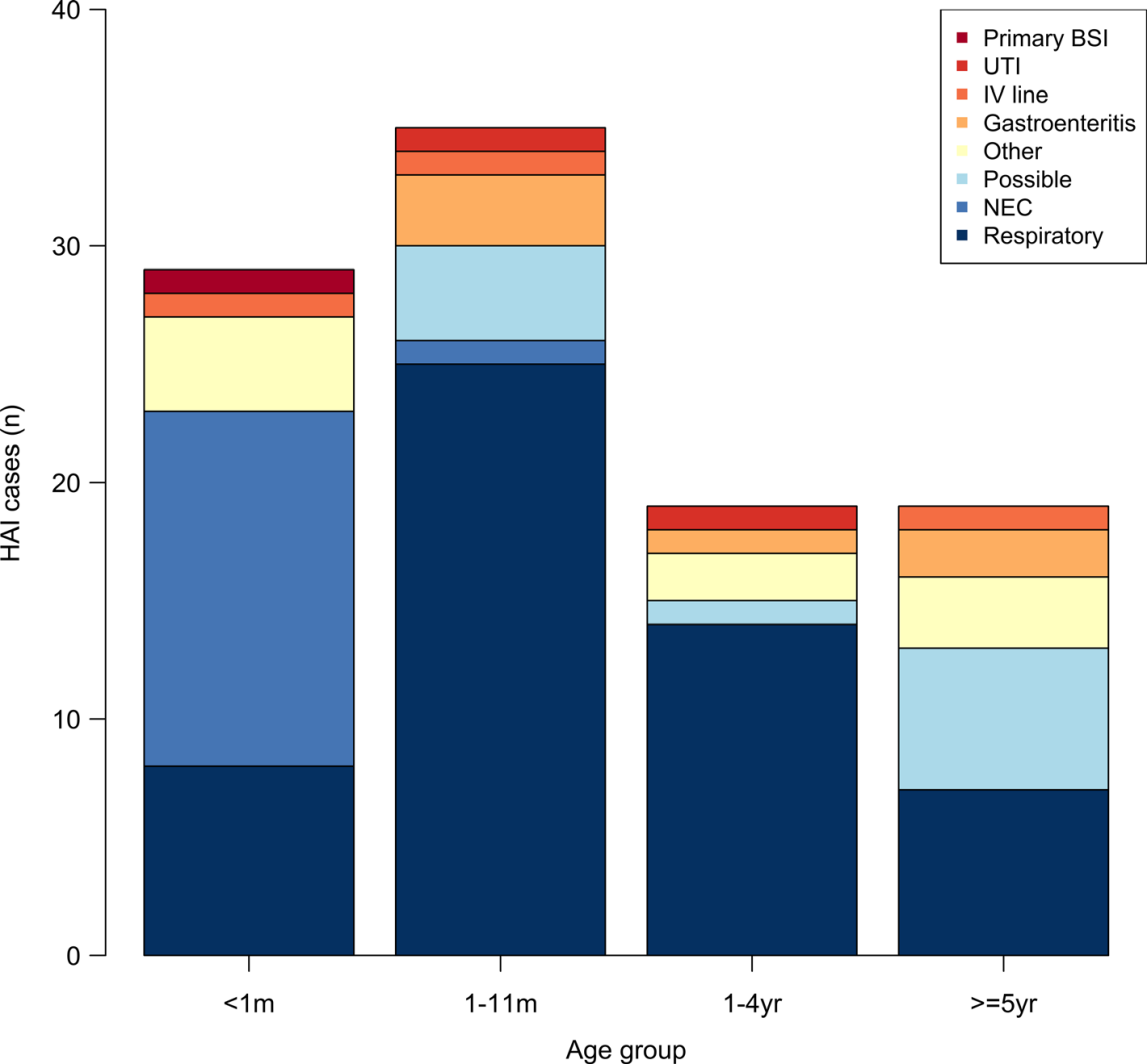
HAI syndrome by age group. *BSI* Blood stream infection, *IV* intravenous, *m* months of age, *n* number of patients, *NEC* necrotizing enterocolitis, *UTI* urinary tract infection, *yr* years of age

The median age of those with an HAI was lower than those without (0.7 vs. 1.8 years, *p* < 0.0001; Table 1) and the HAI syndromes diagnosed varied by age group (Fig. 3). Overall, the most common HAI were respiratory infections: 52.9% (54/102) of cases. These were categorized as hospital acquired pneumonia (29), ventilator associated pneumonia (13), lower respiratory tract infection (8), and upper respiratory tract infection (4). Necrotizing enterocolitis (NEC) was the most common to affect the neonatal age group. Microbiology results revealed 16 HAI cases (15.7%) were associated with significant bacterial isolates from any site, 14 of which were blood cultures. Gram negative organisms numbered 13/16 (81.3%) and overall, third generation cephalosporins were ineffective against 12/16 (75%) clinical isolates (Table 2). Results lead to a change in empirical treatment in 5/16 (31.3%) cases. Third generation cephalosporins made up 18.7% of antimicrobial use in HAI cases, whilst carbapenems accounted for 67.1%.

**Table 1:** Demographic details for all Non-HAI and HAI admissions to Angkor Hospital for Children, 1st January – 31st December 2015

	Non-HAI	HAI	*p*-value
Admissions, n	3,170	93 (102 episodes)	n/a
Age, years (range)	1.8 (0.0–16.0)	0.7 (0.0–15.8)	<0.0001
Gender, % male (male (n): female (n))	57.7 (1830:1340)	65.6 (61:32)	0.1
All-cause, in-hospital mortality rate, % (n)	2.0 (62)	16.1 (15)	<0.0001

*n* number of patients

**Table 2:** Significant bacterial isolates from specimens submitted during work up for suspected HAI

Organism	Sample type	Syndrome	Sensitivity to 3GC	Empirical Rx	Empirical Rx changed
*Acinetobacter baumanii*	ETT	VAP	R	Imi/mero	No
*Acinetobacter baumanii*	B/C	VAP	R	Ceftriaxone	Yes
Coliform^a^	B/C	Gastro	S	Imi/mero	No
*Enterobacter cloacae*	B/C	Primary BSI	R	Imi/mero	No
*Enterobacter cloacae*	B/C	Gastro	S	Imi/mero	No
*Enterobacter cloacae*	B/C	Line	R	Imi/mero	No
*Escherichia coli*	Urine	UTI	R	Imi/mero	No
*Escherichia coli*	B/C & urine	UTI	R	Imi/mero	No
*Escherichia coli Pseudomonas aeruginosa*	B/C	NEC	S Int-R	Imi/mero	No
*Klebsiella pneumoniae*	B/C	HAP	R	Imi/mero	No
*Klebsiella pneumoniae*	B/C	VAP	R	Imi/mero	No
*Moraxella catarrhalis*	B/C	HAP	S	Ceftriaxone	No
*Staphylococcus aureus* (MRSA)	B/C	SSTI	R	Imi/mero	Yes
*Stenotrophomonas maltophilia Enterococcus* species	B/C	NEC	Int-R Int-R	Imi/mero	Yes
*Streptococcus pyogenes*	B/C	SSTI	S	Cloxacillin	Yes
Yeast	B/C	Gastro	Int-R	Ceftriaxone	Yes

*3GC* third generation cephalosporin, *B/C* blood culture, *BSI* blood stream infection, *ETT* endotracheal tube secretions, *Gastro* gastroenteritis, *HAP* hospital-acquired pneumonia, *Imi/mero* carbapenem (imipenem or meropenem), *Int-R* intrinsically resistant, *MRSA* methicillin resistant *Staphylococcus aureus,* *NEC* necrotizing enterocolitis, *R* resistant, *Rx* treatment, *S* sensitive, *SSTI* skin and soft tissue infection, *UTI* urinary tract infection, *VAP* ventilator-associated pneumonia,

^a^This coliform could not be identified to a satisfactory level using the biochemistry short set or bioMerieux API kits available at AHC

HAI were associated with increased mortality. Non-HAI patients had an all-cause in-hospital mortality rate of 2.0% compared to 16.1% for HAI cases (*p* < 0.0001). These infections were deemed to have directly contributed to the deaths of 11 patients, giving an attributable mortality of 11.8%.

Overall, the median LOS for HAI cases was significantly longer than for non-HAI controls: 25 days (interquartile range (IQR) 12–37) compared with 5 days (IQR 3–9; *p* < 0.0001; Table 3). The total cost of treatment for all 102 HAI cases combined was estimated to be $299,608 (Table 3). The direct cost of antimicrobials for the same group combined was $28,472, of which $27,668 (97.2%) was due to carbapenem use.

**Table 3:** Matched LOS and inflation-adjusted HAI cost by age group and admission ward

	Matched cases/controls (n)	Control median LOS (days)	Case median LOS (days)	*p*-value	Extra LOS per HAI (days)	Total LOS due to HAI (days)	Cost per patient-day (US$)	Extra cost per HAI (US$)	Total cost due to HAI (US$)
ICU									
< 1 m	25/50	7	30	<0.0001	23	575	184.5	4,243.5	106,088
1–11 m	15/30	6	32	<0.0001	26	390	184.5	4,797	71,955
1–4 years	6/12	3.5	22	0.003	18.5	111	184.5	3,413.25	20,480
> 5 years	8/16	4.5	36	0.01	31.5	252	184.5	5,843.25	46,746
IPD									
< 1 m	2/4	3.5	37.5	0.1	34	68	130	4,420	8,840
1–11 m	13/26	3.5	17	<0.0001	13.5	175.5	130	1,755	22,815
1–4 years	13/26	3.5	11	<0.0001	7.5	97.5	130	975	12,675
> 5 years	11/22	5	12	0.003	7	77	130	910	10,010

*NICU* cases were considered within the ICU group, *SCBU* cases were considered within the IPD group; *p*-values were derived using the Wilcoxon rank-sum test to compare median LOS

*HAI* Healthcare associated infection, *ICU* intensive care unit, *IPD* in-patient department, *LOS* length of stay, *m* months of age, *n* number of patients, *NICU* neonatal intensive care unit, *SCBU* special care baby unit, *US$* United States dollar, *yr* years of age

During the 12-month surveillance period, average hand hygiene compliance rates at AHC were 81%. The “MicroGuide” app was viewed a total of 4,615 times and a survey of AHC doctors suggested that 96% had used the app during their clinical practice, whilst over half found the app met their needs ‘very’ or ‘extremely well’. PPS data determined that antimicrobial prescriptions were appropriate in 75.4% of all infection cases assessed.

## Discussion

This surveillance demonstrates that accurate HAI incidence data can be produced as part of routine microbiology input in a low-income setting. Few prospective studies have attempted to longitudinally monitor HAI rates in such settings, especially within the pediatric population. These results highlight how simple, affordable measures, such as can be provided by an IPC nurse and an infection doctor, working with ward staff well educated on IPC issues, can result in an HAI incidence of 4.6 per 1,000 patient-days. This is significantly lower than would have been expected from the literature [4, 31] and the trend is downward during the course of the year, although not statistically significant. Nevertheless, HAI cases are avoidable and represent significant costs in terms of broad spectrum antimicrobial use, additional LOS and increased mortality rates.

Murni et al. showed that in resource-limited pediatric settings, a multifaceted intervention including a hand hygiene campaign and the introduction of antimicrobial stewardship can have significant effects on HAI rates and mortality [24]. At AHC the IPC program was established in 2011, with antimicrobial stewardship activities following more recently. Since that time, the HAI attack rate has decreased from 13.8 [25] to the current 3.1 per 100 admissions. The two rates were produced using quite different methodologies and populations, but are nevertheless informative. Ongoing prospective surveillance will bring consistency and allow the impact of future IPC activities to be assessed more accurately.

The introduction of freely available, locally relevant guidelines is a key component of IPC and antimicrobial stewardship [17, 32, 33]. The direct effects of the AHC “MicroGuide” app could not be measured, but it has been widely adopted and utilized by ward clinicians. Antimicrobial prescribing was found to be appropriate in over 75% of cases. The rate of carbapenem use for HAI was high at 67.1%, representing 97.2% of the overall antimicrobial costs, but this is supported by local susceptibility patterns and guidelines [34]. As resistance patterns change and different antimicrobial combinations become available, this practice will continue to be the focus of antimicrobial stewardship efforts, to ensure appropriate use of precious antimicrobials.

According to the literature, around 80% of all HAI are related to medical devices or surgical interventions [2]. However, at AHC urinary and central venous catheters were present in only 9.8 and 2.0% of HAI cases respectively. Limiting the duration of exposure to such medical devices forms part of IPC training at AHC and may have helped maintain the low HAI rates. Future HAI surveillance would benefit further from the collection of data regarding ‘device-days’, to allow comparisons between healthcare settings internationally. These should include ventilator-days, central venous catheter-days and urinary catheter-days and their lack is a limitation of this surveillance, but requires records to be kept for patients throughout the hospital, rather than just the HAI cases monitored here.

Having a dedicated infection team, permanently based on the wards, would likely produce more accurate HAI incidence rates, but may not be possible in many resource-limited settings due to staffing constraints. Major strengths of this work are that it employed a practicable approach, confirmed each HAI case according to strict definitions and involved clinicians in the identification of cases and the collection of clinical data. However, collaboration with the surgical team was limited and accurate data collection was therefore not possible. This is likely to have produced an incomplete picture of the overall HAI incidence. Since surgical site infections are known to be a common cause of HAI [2, 3, 35], working alongside surgical teams must remain a priority wherever possible and the focus of future efforts.

In the context of this surveillance period, it was not possible to link patterns of HAI rates to possible seasonal variations in pathogen prevalence. Limited diagnostics at AHC meant the role of viruses could not be investigated. Viruses are known to contribute to pediatric HAI cases in a way that they do not in adults [11]. It is possible that testing for viruses would have reduced the number of “possible HAI” diagnoses, whilst continuation of the program may yet uncover seasonal patterns, explaining some of the undulation within the incidence curve.

Rates of NEC were found to be high and, after respiratory HAI, represented the second most commonly diagnosed syndrome. The pathogenesis of NEC is incompletely understood, but likely to be multifactorial and limited data exist regarding approaches to reducing its rates [36]. Resistant organisms are quick to colonize areas such as the neonatal unit [37] and difficult to treat once implicated in infections. It remains to be seen whether efforts to improve IPC practices will reduce the chances of colonization with multiply-resistant organisms rather than reduce the number of NEC cases, but this is a question for future research.

Another limitation of this work is the possibility that the incremental human and economic costs of HAI have been overestimated, since confounding or competing risks of adverse outcome and HAI were not accounted for in the analysis. Furthermore, the incremental cost of longer admissions was calculated using the average cost per inpatient day, which does not account for the unequal distribution of costs over the course of an admission. The methods do, however, give a useful estimate of the overall costs of treatment using available data in a setting where more sophisticated models for calculating costs throughout an admission are lacking.

## Conclusions

This report demonstrates that prospective HAI surveillance is possible as part of routine practice in a low-income, pediatric setting. It describes how, in the context of simple IPC and antimicrobial stewardship activities, a relatively low rate of HAI can be maintained. However, despite this low incidence, the overall impact of HAI remains high. The increased mortality rate, cost implications of additional carbapenem use and extra LOS in hospital are all compelling reasons to pursue cost effective and sustainable approaches to HAI reduction. Ongoing, prospective surveillance will allow the effectiveness of these approaches to be closely monitored and will inform ways in which further reductions to HAI rates can best be achieved.

## Additional file

Additional file 1: HAI_case_definitions. (DOCX 23 kb)

## Abbreviations

AHC: Angkor Hospital for Children
CDC: Centers for disease control and prevention
CI: Confidence interval
CLSI: Clinical & Laboratory Standards Institute
CME: Continued medical education
HAI: Healthcare associated infection
ICU: Intensive care unit
INICC: International nosocomial infection control consortium
IPC: Infection prevention and control
IPD: In-patient department (medical wards)
IQR: Interquartile range
LOS: Length of stay
NEC: Necrotizing enterocolitis
NICU: Neonatal intensive care unit
PPS: Point prevalence survey
SCBU: Special care baby unit
VAP: Ventilator associated pneumonia
WHO: World Health Organization
