# Supplementary material for: Prospective surveillance of healthcare associated infections in a Cambodian pediatric hospital
Source: Antimicrob Resist Infect Control. 2017 Jan 23;6:16. doi: 10.1186/s13756-017-0172-5 (PMC5260112; doi:10.1186/s13756-017-0172-5)
Supplement: Additional file 1: — HAI_case_definitions. (DOCX 23 kb) [file 13756_2017_172_MOESM1_ESM.docx]

| **Syndrome** | **Definition** | **Comment** |
| --- | --- | --- |
| Bloodstream infection | **Primary**  Definite pathogen from ≥1 blood culture ***AND*** not related to infection at another site  ***OR***  >1 year old: fever (≥38°C), chills or prolonged CRT (>2 sec) ***AND*** a commensal organism (e.g. coagulase-negative staphylococcus) from ≥2 blood cultures ***AND*** not related to infection at another site  ≤1 year old: fever (≥38°C), hypothermia (<36°C), apnoea, or bradycardia (<100 beats/min) ***AND*** a commensal organism (e.g. coagulase-negative staphylococcus) from ≥2 blood cultures ***AND*** not related to infection at another site | (See complete list of common commensals at <http://www.cdc.gov/nhsn/XLS/master-organism-Com-Commensals-Lists.xlsx>)  CDC definition uses hypotension rather than prolonged CRT (but does not define further) |
|  | **Secondary**  Positive blood culture, as defined for primary BSI, in the presence of an infection at another site | Report the clinical syndrome and also report the secondary BSI |
| Gastroenteritis | Acute onset of diarrhoea (liquid stools for >12 hours) with or without vomiting or fever (≥38°C) ***AND***  no likely non-infectious cause (e.g. lactose intolerance)  ***OR***  No diarrhoea ***AND*** at least two of: fever (≥38°C), nausea*, vomiting*, abdominal pain* or headache* ***AND*** an enteric pathogen identified by microscopy, culture, antigen / antibody test, or PCR |  |
| Necrotising enterocolitis | At least one clinical sign ***AND*** one imaging finding  Clinical: bilious aspirate, vomiting, abdominal distention, occult or gross blood in stool (without rectal fissure)  Imaging: pneumatosis intestinalis, portal venous gas, pneumoperitoneum  ***OR***  Surgical NEC: extensive bowel necrosis (>2cm) OR surgical evidence of pneumatosis intestinalis +/- intestinal necrosis | Infant ≤1 year only |
| Respiratory infection | **URTI**  >1 year old: at least two of: fever (≥38°C), sore, red, or purulent throat*, cough*, or hoarseness*  ≤1 year old: at least two of: fever (≥38°C), hypothermia (<36°C), apnoea*, bradycardia (<100 beats/min) *, nasal discharge* or purulent exudate in throat*  ***AND***  Organisms culture from the specific site or blood ***OR*** positive respiratory secretion pathogen IFA / PCR / RDT ***OR***  clinician diagnosis of URTI |  |
|  | **LRTI – non-pneumonia**  No clinical or radiological evidence of pneumonia  ***AND***  >1 year old: at least two of: fever (≥38°C), cough*, new or increased sputum production*, rhonchi or wheeze*  ≤1 year old: at least two of: fever (≥38°C), cough*, apnoea*, bradycardia (<100 beats/min) *, new or increased sputum production*, rhonchi or wheeze*, or respiratory distress* | CDC definition requires a positive culture (bronchoscopy or deep tracheal aspirate) , IFA / PCR / RDT on respiratory secretions, or serology |
|  | **Pneumonia**  CXR shows new or progressive infiltrate ***OR*** consolidation ***OR***  cavitation ***OR***  pneumatocoeles (≤1 year old)  ***AND***  ≤1 year old: worsening gas exchange ***AND*** at least three of:   - Temperature instability - Bradycardia (<100 beats/min) or tachycardia (>160 beats/min) - Leukopenia (<4.0 x 10^9^/ml) or leucocytosis (>15.0 x 10^9^/ml + left shift) - New onset purulent sputum or increase respiratory secretions / suctioning requirements - Cough - Apnoea or respiratory distress (tachypnoea, nasal flaring, grunting, chest indrawing) - Crepitations or wheeze on auscultation   >1 year old: at least three of:   - Fever (≥38°C),or hypothermia (<36°C) - Leukopenia (<4.0 x 10^9^/ml) or leucocytosis (>15.0 x 10^9^/ml) - New onset purulent sputum or increase respiratory secretions / suctioning requirements - New or worsening cough or respiratory distress (dyspnoea or tachypnoea) - Crepitations or wheeze on auscultation - Worsening gas exchange | See Appendix 1 for VAP definition  CDC definitions also include pathogen-specific laboratory results  CDC define infant tachycardia as <170 beats/min. 160 is used to be consistent with the AHC sepsis protocol  Worsening gas exchange = O_2_ desaturations (<94%), increased oxygen requirements, or increased ventilator demand |
| Surgical site infection | **Superficial**  Purulent drainage from the superficial incision  ***OR***  Organisms isolated from an aseptically obtained culture from the superficial incision  ***OR***  Deliberate opening of the superficial incision by the surgeon ***AND*** pain, tenderness, redness, localised swelling, or heat  ***OR***  Diagnosis of superficial SSI by the surgeon | CDC definition includes those occurring within 30 days of the surgical procedure. The AHC system will only capture in-hospital infections |
|  | **Deep**  Purulent drainage from deep incision  ***OR***  Spontaneous dehiscence or deliberate opening by the surgeon ***AND*** fever (≥38°C), pain, or tenderness  ***OR***  Abscess involving the deep incision |  |
|  | **Organ/space**  Purulent drainage from a drain inserted into the organ/space  ***OR***  Organisms isolated from an aseptically obtained culture from the organ space  ***OR***  Abscess involving the organ/space |  |
| Urinary tract infection | **Catheter-related**  Indwelling urinary catheter in place for >2 days (day of device placement = day 1), and catheter was in place on the date of event  ***AND***  >1 year old: At least one of: fever (>38°C), suprapubic tenderness*****, or renal angle pain or tenderness*****  ≤1 year old: fever (≥38°C), hypothermia (<36°C), apnoea*, bradycardia (<100 beats/min) *, dysuria*, lethargy*, or vomiting*  ***AND***  Positive urine culture of ≥10^5^ cfu/ml with no more than two species of microorganisms. ***OR*** a single species of ≥10^4^ cfu/ml | CDC has many more sub-divisions based on catheter duration and microscopic findings |
|  | **Non-catheter related**  Indwelling urinary catheter not in place for >2 days (day of device placement = day 1) or on the day of the event  ***AND***  >1 year old: At least one of: fever (>38°C), urgency*, frequency*, dysuria*, suprapubic tenderness*****, or renal angle pain or tenderness*****  ≤1 year old: fever (≥38°C), hypothermia (<36°C), apnoea*, bradycardia (<100 beats/min) *, dysuria*, lethargy* or vomiting*  ***AND***  Positive urine culture of ≥10^5^ cfu/ml with no more than two species of microorganisms. ***OR*** a single species of ≥10^4^ cfu/ml |  |
| Vascular-line associated infection | Fever (≥38°C), pain*, redness*, or heat* at a vascular line site  ***OR***  Purulent drainage at a vascular line site | CDC definition includes semi-quantitative line tip culture |
| Other | Clear clinical syndrome (e.g. osteomyelitis) commencing >2 days / 48 hours after admission | Refer to CDC/NHSN Surveillance Definitions for Specific Types of Infections (Centers for Disease Control; 2014) to confirm case definitions for uncommon HCAI |
| Possible HCAI | Clinician diagnosis is HCAI (or “HAI” or “nosocomial infection”) ***AND***  antimicrobials have been started/changed to reflect this diagnosis ***BUT*** none of the HCAI syndrome case definitions are met |  |

*****With no other recognised cause

**Appendix 1: AHC diagnostic criteria for ventilator-associated pneumonia**

**1. Radiologic signs**: CXR with at least **one** of the following*:

- New or progressive and persistent infiltrate
- Consolidation
- Cavitation
- Pneumatoceles (in infants ≤ 1 year)

*should have 2 separate CXRs which show above findings, if >72hrs before previous CXR since other complications of intubation can cause abnormal findings (e.g. atelectasis, pulmonary edema, alveolar hemorrhage, etc.)

***AND***

**2. Clinical signs**: at least **three** of the following:

- Temperature >38°C or <36.0°C with no other recognized cause
- Leukopenia (leukocyte count <4.0 × 10^9^ cells/L) or leukocytosis (leukocyte count >15.0 × 10^9^ cells/L) or elevated CRP
- New onset of purulent sputum or increased respiratory secretions or increased suctioning requirements
- New onset of worsening cough, or dyspnea, or tachypnea, or apnea
- New rales/crackles or bronchial breath sounds
- Worsening gas exchange (e.g. O_2_ desaturations, increased oxygen requirements, or increased ventilation demand)
- Positive microbiology of any of the following:
  - Positive ETT culture with either moderate amount WBCs or at least moderate amount of bacteria (10^5^ cfu/mL)
  - Positive blood culture not related to any other infection
  - Positive pleural fluid culture

*NOTE: There is no minimum period of time that the patient must have been ventilated in order to consider a new pneumonia to be ventilator-associated*
